# Proposed protocol for treatment of severe periodontitis without platelet transfusion in patients with aplastic anemia: a case report

**DOI:** 10.1186/s13256-021-03170-0

**Published:** 2021-12-10

**Authors:** Satoru Morikawa, Kazuya Watanabe, Satoshi Usuda, Yoko Miyashita, Taneaki Nakagawa

**Affiliations:** 1 Department of Dentistry and Oral Surgery, Keio University School of Medicine, 35 Shinanomachi, Shinjuku-ku, Tokyo, 160-8582 Japan; 2 Watanabe Orthodontic Office, 1-11-26-2F Kichijoji-honcho, Musashino-shi, Tokyo, 180-0004 Japan

**Keywords:** Dentistry, Oral medicine, Hematology, Severe periodontitis, Aplastic anemia

## Abstract

**Background:**

Aplastic anemia is an intractable disease characterized by pancytopenia, susceptibility to infection, and difficulty in achieving hemostasis. In patients with severe periodontal disease and aplastic anemia, spontaneous bleeding from the gingival tissue due to thrombocytopenia and during brushing is common, which may further exacerbate dental issues. Comprehensive periodontal treatment for patients with aplastic anemia is highly challenging and requires collaboration with a hematologist. Here, we discuss the case of a patient with aplastic anemia and severe periodontitis who was successfully treated in collaboration with our hematology department.

**Case presentation:**

A 36-year-old Japanese woman with chief complaints of spontaneous gingival bleeding, pain, and increasing tooth mobility consulted our department. She had developed pancytopenia at age 11 years and was later diagnosed with aplastic anemia, making her susceptible to infection due to leukopenia. The results of the initial periodontal examination led to a diagnosis of severe generalized periodontitis (generalized stage IV grade C periodontitis) caused by leukopenia and poor oral hygiene. We adopted a comprehensive treatment plan, including invasive dental procedures. The patient exhibited no postoperative bleeding due to aplastic anemia-induced thrombocytopenia and experienced a good outcome.

**Conclusions:**

Both physicians and dentists should be aware that immunocompromised patients with aplastic anemia are at risk of developing severe periodontitis with severe alveolar bone resorption if the condition is combined with poor oral hygiene. Even in the presence of aplastic anemia, patients with severe periodontitis can undergo comprehensive dental treatment, including dental extraction and periodontal surgery, if bleeding and susceptibility to infection are controlled. This requires the cooperation of the patient and hematologists and can ultimately contribute to improving the patient’s quality of life.

## Background

Aplastic anemia (AA) is an intractable hematologic disease characterized by empty bone marrow that results in pancytopenia with decreased levels of platelets, white blood cells, and erythrocytes [1, 2]. There are three possible pathophysiological mechanisms of AA: chemical and physical damage, immune destruction, and constitutional genetic defects. For these reasons, AA is characterized by the replacement of hematopoietic cells with fat [1]. Pancytopenia causes symptoms of infection such as fever and cough due to a decrease in white blood cells; palpitations, shortness of breath, and fatigue due to a decrease in red blood cells; and thrombocytopenic hemorrhagic (especially epistaxis and gingival bleeding). Gingival bleeding can make patients hesitant to brush their teeth, resulting in poor oral hygiene and subsequent periodontitis. Periodontal treatment should be performed in coordination with a hematologist, especially for severe periodontitis requiring prolonged treatment. Peripheral blood tests can also help to identify leukopenia, thrombocytopenia, and anemia.

Patients with AA exhibiting leukopenia are at a high risk of developing systemic infection due to neutropenia. Previous research has indicated that fungal infections and bacterial sepsis are the most frequent sources of death in patients with AA [2]. Furthermore, in patients with thrombocytopenia, initial periodontal treatment, including subgingival scaling and root planning (SRP), may cause uncontrolled postoperative bleeding (POB). POB has been defined as “that which: (1) continues beyond 12 hours; (2) causes the patient to call or return to the dental practitioner or to the emergency department; (3) results in the development of a large hematoma or ecchymosis within the oral soft tissues; or (4) requires a blood transfusion” [3].

Practitioners have considered a platelet count ≥ 50,000/μl as necessary to ensure the safety of invasive dental procedures. In addition, platelet transfusions are recommended for patients with a platelet count < 50,000/μl prior to invasive dental procedures [4, 5] and for patients with a platelet count < 30,000/μl prior to SRP [6]. On the other hand, platelet transfusions can lead to the emergence of antiplatelet antibodies and viral infections, and these transfusions can be costly, making it best to limit their use. Here, we present the case of a patient with AA, who was effectively treated for severe periodontitis via comprehensive periodontal therapy, in collaboration with our hematology department.

## Case presentation

The patient was a 36-year-old Japanese woman who consulted our department regarding spontaneous gingival bleeding, pain, and increasing tooth mobility. Additionally, she was aware of root exposure and protrusion of her maxillary anterior teeth. She had developed pancytopenia at age 11 years and was subsequently diagnosed with AA. Although she was treated with immunosuppressive therapy, hematopoietic growth factor, and a gonadotropin-releasing hormone agonist to stop menstruation, pancytopenia did not go into remission. She was also receiving whole blood transfusions approximately once per month. At approximately 20 years of age, her symptoms due to pancytopenia stabilized, and she was followed up in the hematology department. Her white blood cell count was approximately 1000–1500/µl, and her platelet count was low (approximately 15,000–20,000/µl), without further increase. Since her late teens, the patient had experienced spontaneous gingival bleeding and pain. However, previously, she had only been kept under observation and given oral hygiene instructions; she had not undergone active periodontal treatment due to difficulty in achieving hemostasis.

### Investigations

**Fig. 1:**
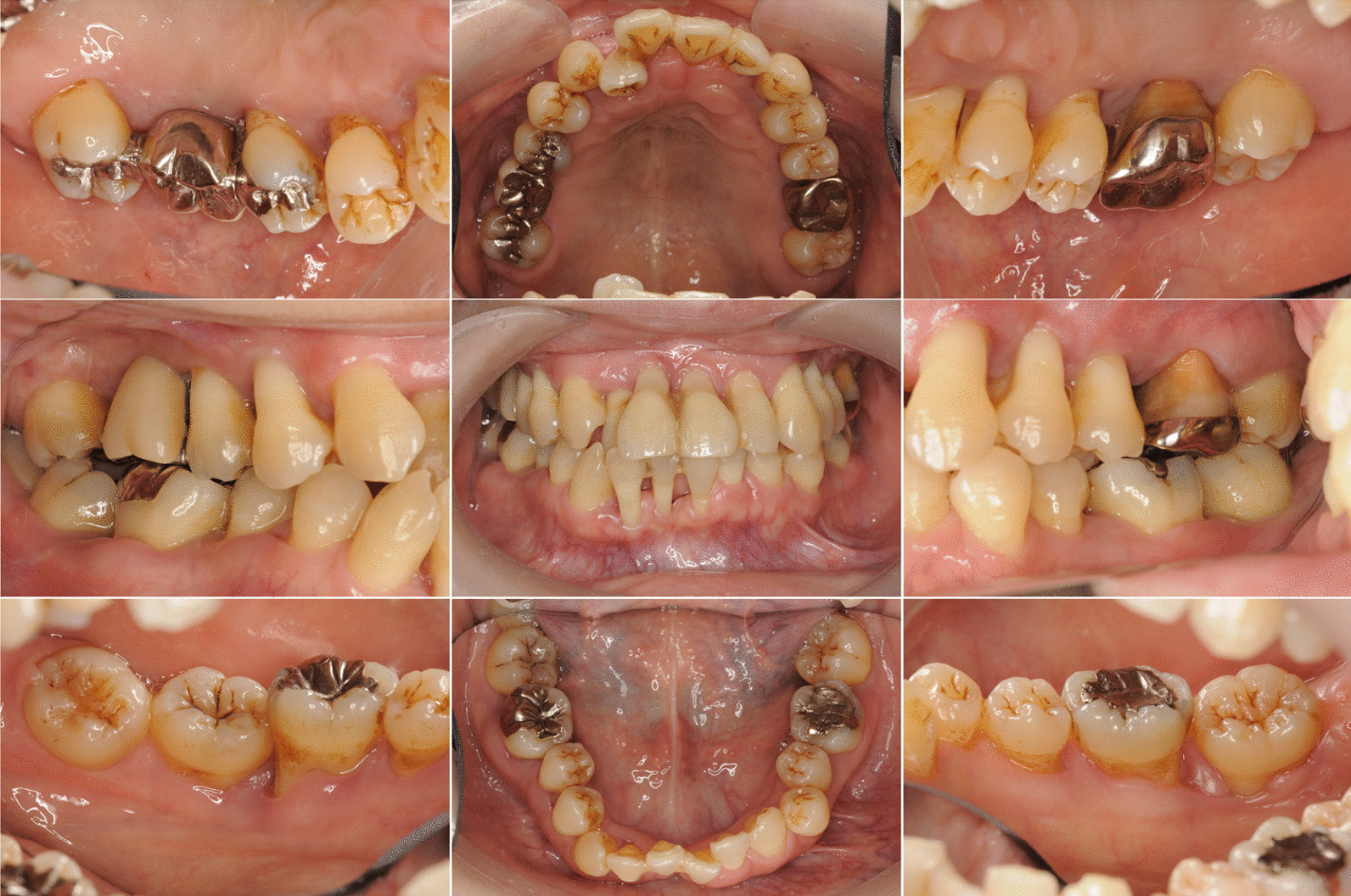
Clinical appearance before treatment. Gingival edema and recession can be observed with pathological tooth movement in the maxillary and mandibular regions

**Fig. 2:**
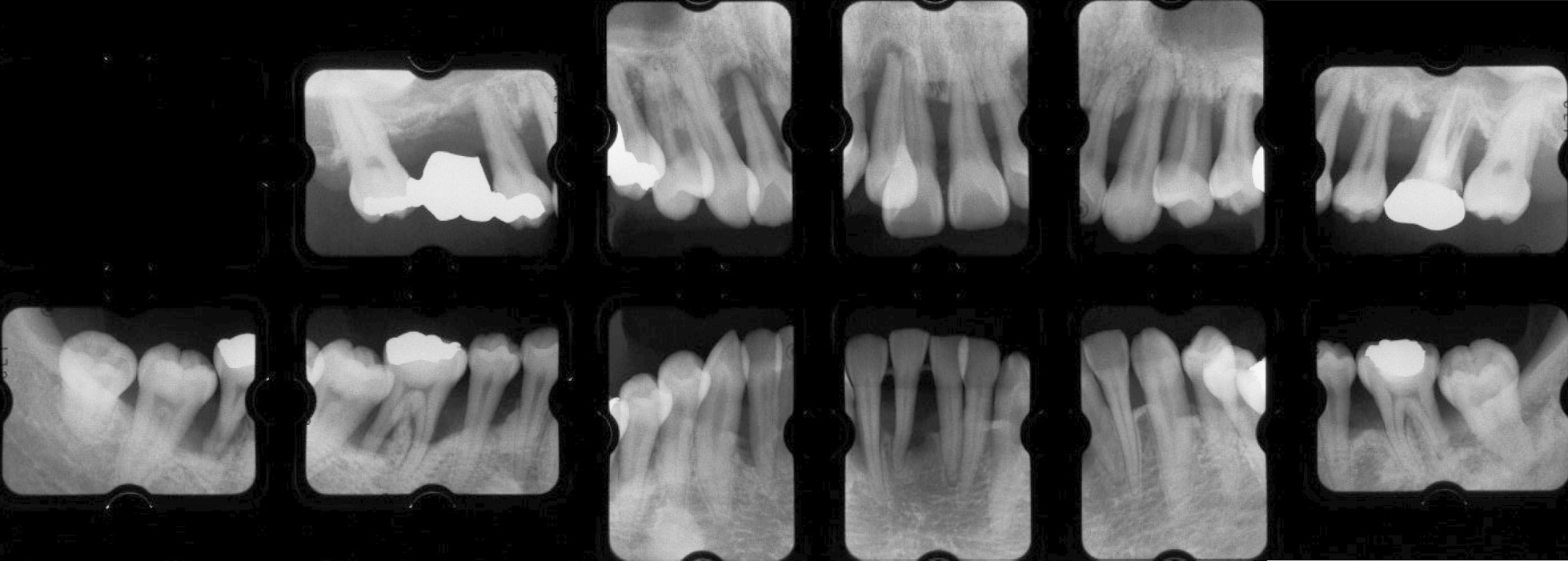
Dental periapical radiography reveals generalized horizontal and vertical bone loss

We performed oral and periodontal examinations to establish a diagnosis and treatment plan. Tooth and soft tissue assessments were conducted (Fig. 1), and periodontal destruction was evaluated by measuring the probing pocket depth (PPD) at six points around each tooth. We performed dental periapical radiography to assess the degree of alveolar bone loss, which was severe, especially in relation to the patient’s age (Fig. 2). We observed deep periodontal pockets with PPDs ranging from 4 to 11 mm. The periodontal inflamed surface area (PISA) was 3102.1 mm^2^ [7]. The results of the initial periodontal examination led to a diagnosis of severe generalized periodontitis (generalized stage IV grade C periodontitis) [8].

The patient underwent general peripheral blood tests, which revealed significantly decreased platelet, leukocyte, and neutrophil levels, as indicated in Table 1.

**Table 1:** Blood test results

	Platelet count (× 10^3^/μl)	White blood cell count (× 10^3^/μl)	Absolute neutrophil count (× 10^3^/μl)	Red blood cell count (× 10^6^/μl)	Tooth	Platelet transfusion	Local hemostasis measures
Scaling	43–47	3.2	1888	4.07	All teeth	−	Pressure hemostasis
SRP (first)	27–38	1.5–3.4	750–2516	4.05–4.24	All teeth	−	Pressure hemostasis
Periodontal surgery (PPD > 6 mm)	48 After platelet transfusion	0.5	340	4.35	26, 27, 37	+	Suture/pressure hemostasis
Periodontal surgery (PPD > 6 mm)	17 After platelet transfusion	1.4	1078	3.44	15, 23	+	Not applicable
FMD (second SRP)	21–23	1.3–1.4	420–429	3.65–3.86	11, 13–15, 17, 21–25, 33–36, 42, 45–48	−	Pressure hemostasis
Extraction	32	1.5	825	4.2	12	+	Suture/pressure hemostasis
Extraction	49 After platelet transfusion	0.4		3.54	26, 41	+	Suture/pressure hemostasis

*FMD* full-mouth disinfection, *PISA* periodontal inflamed surface area, *PPD* probing pocket depth, *SRP* scaling and root planning.

+ Patient received platelet transfusion

− Patient did not receive platelet transfusion

### Treatment

**Fig. 3:**
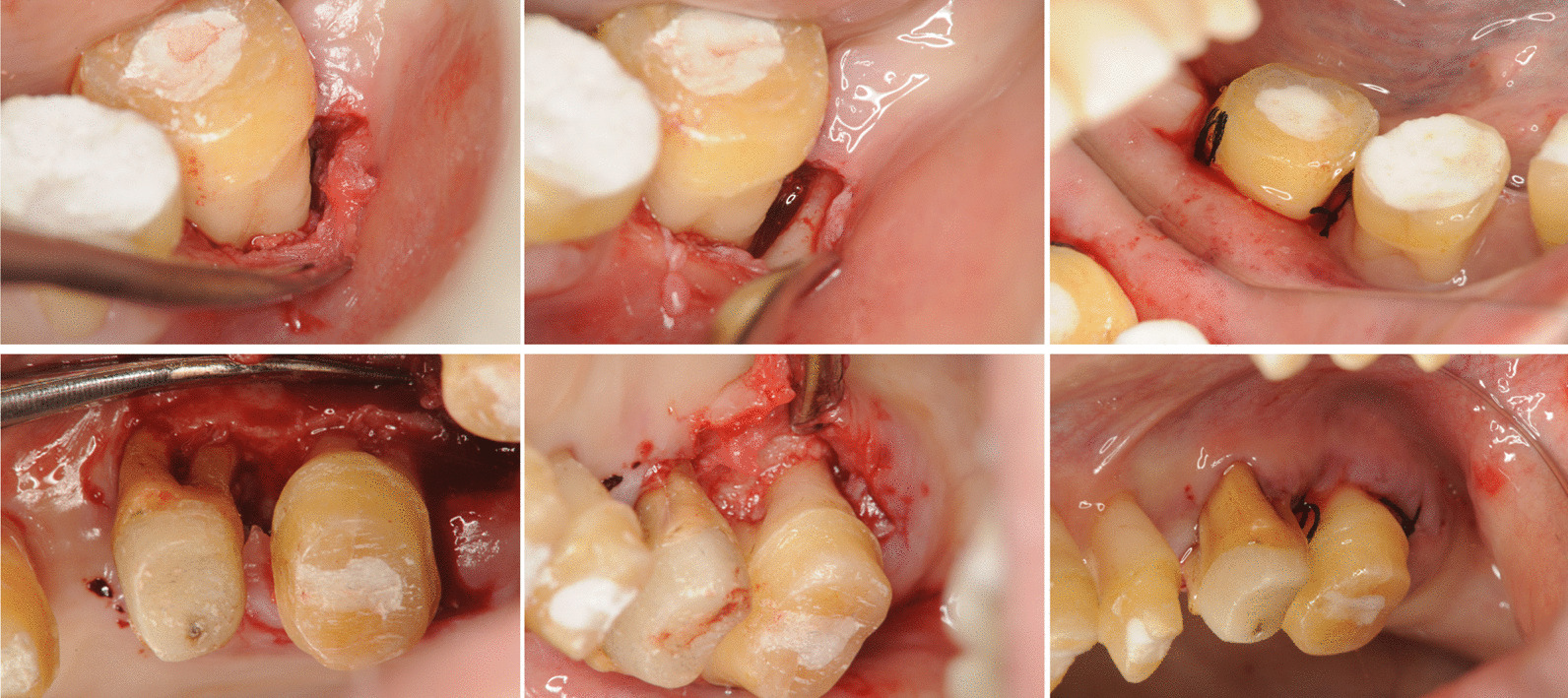
Intraoral pictures of the surgical sites. No persistent intraoperative or postoperative bleeding was observed

**Fig. 4:**
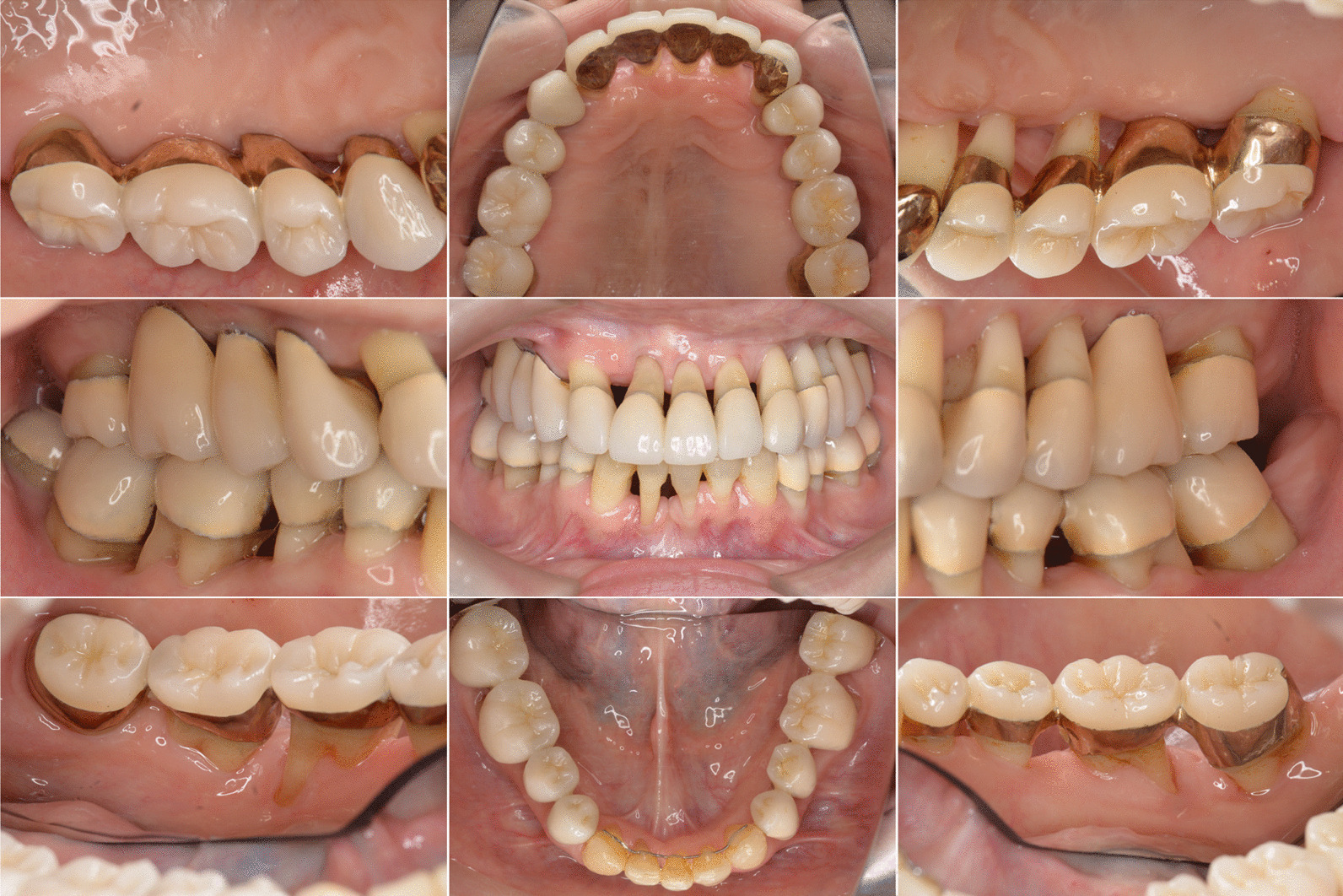
Intraoral clinical appearance after orthodontic and prosthetic treatment. There are no signs of inflammation

**Fig. 5:**
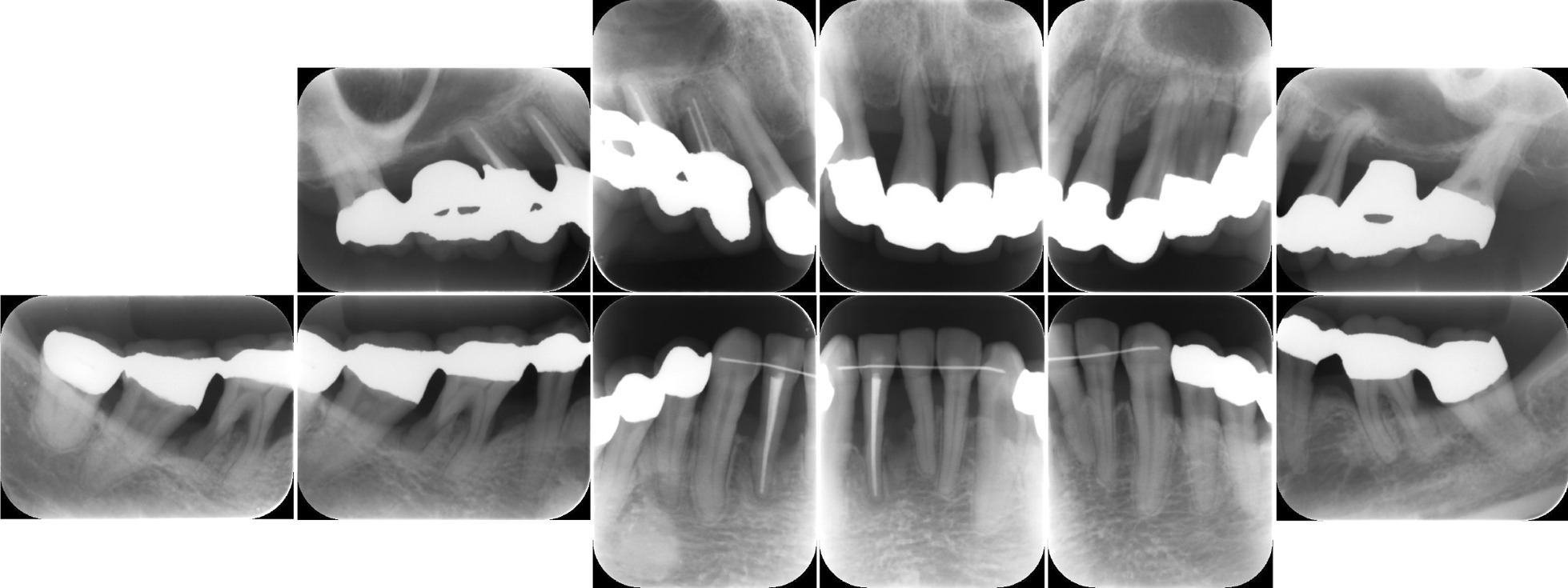
Dental periapical radiography. There are no signs indicating progression of bone resorption, and alveolar bone gain could be observed on the mesial aspect of tooth 17, the distal aspect of tooth 35, and the distal aspect of tooth 42 without bone grafting procedures

We provided the patient thorough oral hygiene instructions and conducted nonsurgical periodontal therapy for a few teeth at a time, which included assessment of gingival bleeding. We also performed blood tests to confirm reliable local hemostasis. We observed improvements in her inflammation score after nonsurgical periodontal therapy, which included subgingival debridement. During the basic periodontal treatment, her absolute neutrophil count ranged from 750 to 2516/µl. Following SRP, we delivered minocycline ointment to the treated periodontal pockets to eliminate periodontal disease-related bacteremia. We administered antimicrobial prophylaxis only when the absolute neutrophil count was ≤ 2000/µl. Scheduled sites for periodontal surgery were 15, 23, 26, 27, and 37 (Federation Dentaire Internationale System) with more than 6 mm of residual PPD after nonsurgical periodontal therapy. We administered antibacterial prophylaxis for leukopenia and prophylactic platelet transfusion for thrombocytopenia, in coordination with the department of hematology. Platelet transfusions were performed on the day before and on the day of periodontal surgery. We performed periodontal surgery after confirming that her platelet count had increased (Table 1). We conducted thorough surgical debridement at sites 26, 27, and 37 after confirming that the platelet count had reached 48,000/μl. We observed no POB (Fig. 3). During periodontal surgery and tooth extraction, leukopenia was observed whether the neutrophil count was above or below 2000/μl, and prophylactic administration of antimicrobial agents (oral amoxicillin) was selected to prevent postsurgical site infection and bacteremia, based on the results of a network meta-analysis [9]. However, we were unable to confirm an increase in platelet count after platelet transfusion prior to periodontal surgery at sites 15 and 23. After preparing acrylic splints for hemostasis at teeth with a moderate probing depth after the initial SRP (11, 13, 14, 17, 21, 22, 24, 25, 33–36, 42, 45–48), we did not proceed with periodontal surgery at sites 15 and 23 and instead performed full-mouth disinfection (FMD) with an antimicrobial agent. To reduce the number of invasive procedures and prevent POB, bacteremia, and septicemia due to thrombocytopenia and leukopenia, we considered FMD the appropriate protocol for management of the remaining periodontally compromised teeth by removing the source of infection via SRP. During FMD, sitafloxacin (100 mg/day) was administered for 7 days to prevent fever and septicemia/bacteremia, which is a potential complication of FMD [10]. A reevaluation revealed that the periodontal inflammation had been controlled through nonsurgical and surgical periodontal treatment. To correct the occlusal trauma caused by tilting of teeth due to a high degree of bone resorption with pathological migration, we planned orthodontic treatment followed by prosthetic rehabilitation. Cephalometric analysis of the orthodontic treatment revealed that extraction of the mandibular anterior teeth in a plexiform state was necessary to move the mandibular anterior teeth lingually, and the tooth with the most significant bone resorption (site 41) was extracted. Furthermore, the root of 26 had fractured after periodontal treatment and required extraction. After platelet transfusion, we confirmed that the platelet count had reached 49,000/μl and extracted teeth 41 and 26 (Table 1). Following the completion of orthodontic treatment, we observed no recurrent findings of periodontal disease. We then proceeded to restore occlusal function using resin–ceramic hybrid facing connected crowns (13–23, 34–38, and 44–48) to obtain a fixed effect, in combination with resin–ceramic hybrid facing bridges (14–17 and 24–27) (Fig. 4). Such treatment also helped to reduce tooth mobility caused by severe alveolar bone resorption. Furthermore, our findings confirmed that alveolar bone resorption had not progressed since the initial examination (Fig. 5). Alveolar bone gain was also observed in relation to teeth 17, 35, and 42 without bone grafting procedures.

### Outcome and follow-up

Compared with the initial examination, the O’Leary plaque control record decreased from 34.8% to 16.0%, bleeding on probing decreased from 134 sites (79.8%) to 13 sites (8.7%), the average PPD decreased from 5.6 to 2.3 mm, and PISA decreased from 3102.1 to 95.0 mm^2^. Currently, the patient is being followed up with supportive periodontal therapy and exhibits a stable course with no recurrence of periodontal disease.

## Discussion

The present case raises two important points. First, in young people, pancytopenia caused by AA accompanied by poor oral hygiene can trigger severe early-onset periodontitis. Control of the disease can be difficult when both are present. However, close medical and dental cooperation can help restore the patient’ quality of life. Second, when the platelet count is 20,000 μl, complete SRP can be performed without POB or the need for platelet transfusion. In addition, prophylactic platelet transfusion before periodontal surgery or tooth extraction may not be necessary if local hemostatic measures are taken.

The purpose of periodontal therapy for AA is to prevent and manage the most common complications, which include infection and hemorrhage. Invasive dental procedures, including periodontal treatment, can be life-threatening for patients suffering from severe AA. Therefore, the dentist should prescribe hematological assessment in coordination with a physician to evaluate leukocyte and platelet counts before instituting any therapy. In patients with AA, it is also important to monitor the white blood cell count, especially the neutrophil count, as a decreased white blood cell count can lead to bacteremia and sepsis. A previous prospective study reported that patients with periodontal infections may experience bacteremia more often than periodontally healthy controls during neutropenia [11]. In the present case, our patient’s neutrophil count was ≤ 2000/μl at the initial visit, which indicated a weak immune response. The first step of initial periodontal treatment involved oral hygiene instruction and supragingival scaling to avoid the risk of infection and reduce the bacterial load. Subsequently, we conducted subgingival SRP with frequent saline irrigation and then delivered minocycline ointment to periodontal pockets when the neutrophil count was within the range of 750–2500/μl. Previous research suggests that antibiotics should be administered prior to invasive dental treatment if the neutrophil count is less than 2000/μl [12]. Accordingly, we treated our patient with oral amoxicillin to prevent postprocedural bacteremia when her neutrophil count was less than 2000/μl.

Previous reports have suggested that local hemostasis is possible in patients undergoing oral surgery when platelet counts are approximately 50,000/μl or more [13]. However, a recent systematic review provided no evidence to support the need for a platelet count of ≥ 50,000/μl to ensure the safety of invasive dental procedures in patients with thrombocytopenia [14]. Furthermore, in this review article, the effectiveness of platelet transfusion for hemostasis could not be determined. Although a platelet count of 50,000/μl has long been used to ensure hemostasis during invasive dental procedures [15], this claim has never been substantiated by scientific evidence. The abovementioned systematic review demonstrated that platelet counts did not differ between patients with POB (38,143/μl) and those without POB (38,820/μl). In the present case, we performed the first round of subgingival SRP when the platelet count was within the range of 27,000–38,000/μl, and the second round (FMD) when the platelet count was within the range of 21,000–23,000/μl. Notably, we observed no POB. This is significant in that platelet transfusion may induce refractoriness and increase costs to the healthcare system [16]. However, in the present case, we also performed prophylactic platelet transfusion during tooth extraction and periodontal surgery, although the platelet count did not rise even after platelet transfusion in one instance. Thus, the present case suggests that, when local measures such as suturing and acrylic splints are applied in collaboration with a hematologist, prophylactic platelet transfusion may not be necessary in patients with thrombocytopenia.

Our case also illustrates a potential approach for handling platelet and leukocyte counts during basic periodontal treatment and periodontal surgery. Namely, if the neutrophil count is less than 2000/µl, prophylactic antibiotics should be administered to treat leukopenia. For invasive dental procedures (including SRP, tooth extraction, and periodontal surgery) in patients with a platelet count ≤ 50,000, platelet transfusion may not be necessary to manage POB if adequate local measures (for example, suturing, acrylic splints) are taken in consultation with hematologists. Indeed, the present case indicates that it is important to perform the complete basic periodontal treatment without platelet transfusion when the platelet count is around 20,000/μl.

In this study, the second round of SRP involved FMD with sitafloxacin for the following reasons: (1) to minimize the number of invasive treatments that might have caused POB; (2) to prevent the development of bacteremia or sepsis following SRP as sitafloxacin administration has been described as effective for periodontopathic bacteria [17, 18]; and (3) because a previous meta-analysis and systematic review disclosed that FMD in conjunction with antimicrobial treatment can improve microbiological and clinical outcomes in patients with severe periodontitis [19]. In other words, elimination of the source of infection and complete resolution of the infection with antibiotics can prevent recurrence of the disease with subsequent maintenance or supportive periodontal therapy. The present case highlights the suitability of this protocol for patients with blood disorders involving leukopenia/thrombocytopenia and concurrent severe periodontitis. However, further studies and case reports are required to verify this assumption.

AA-induced pancytopenia plus poor oral hygiene may contribute to the onset of periodontitis at an early age, and AA-induced hemostatic difficulties may delay early periodontal interventions. Both doctors and dentists should be aware of the possibility of both diseases (leukopenia and rapid progressive periodontitis) as well as the need to assess and intervene as early as possible. Since AA is associated with pancytopenia, it is possible that our patient was susceptible to periodontal disease from the age of 11 years when she originally developed AA due to the increased risk of infection caused by leukopenia. The patient also experienced spontaneous gingival bleeding due to thrombocytopenia and during brushing. The patient reported refraining from brushing owing to fear of bleeding. This suggests that plaque buildup caused by poor oral hygiene and lack of proper brushing instructions contributed to her condition. In addition, the combination of her immunocompromised state and plaque accumulation may have triggered severe periodontitis. Patients with AA, especially those who develop AA during childhood, may be at risk for severe alveolar bone resorption and significant impairment of masticatory function at an early age due to early tooth loss. In other words, the number of remaining teeth and the degree of alveolar bone resorption may differ depending on when AA develops and whether therapeutic interventions are administered. When physicians treat pancytopenic diseases, they should not only control the primary disease, but should also work closely with medical and dental staff to avoid missing oral symptoms. Dentists must also be aware of the oral manifestations of pancytopenia and refer patients to specialists and institutions when necessary.

## Conclusions

Pancytopenia accompanied by poor oral hygiene condition can induce severe periodontitis. When both diseases are present, close medical and dental cooperation is required to prevent life-threatening events and improve the patient’s quality of life. The present case suggests that, when local hemostatic measures have been adequately applied, a platelet count of 20,000 is sufficient to perform invasive dental procedures without the need for platelet transfusions. However, further validation and additional case reports are required to verify this assumption. Even if the patient has AA and severe chronic periodontitis, comprehensive dental treatment including tooth conservation, extraction surgery, orthodontic treatment, and prosthetic treatment is possible by controlling the primary disease in cooperation with hematology.

## Data Availability

All data generated or analyzed during this study are included in this published article. The dataset created during and/or analyzed during this case are available from the corresponding author of reasonable request.

## Abbreviations

AA: Aplastic anemia
FMD: Full-mouth disinfection
POB: Postoperative bleeding
PPD: Probing pocket depths
SRP: Scaling and root planning
